# Vitamin C Protects Porcine Oocytes From Microcystin-LR Toxicity During Maturation

**DOI:** 10.3389/fcell.2020.582715

**Published:** 2020-10-08

**Authors:** Xue Zhang, Changyin Zhou, Weijian Li, Juan Li, Wangjun Wu, Jingli Tao, Honglin Liu

**Affiliations:** Department of Animal Genetics, Breeding and Reproduction, College of Animal Science and Technology, Nanjing Agricultural University, Nanjing, China

**Keywords:** MC-LR, vitamin C, oocyte, meiosis, ROS

## Abstract

Microcystin-leucine arginine (MC-LR) is the most toxic cyanotoxin found in water bodies. Microcystins are produced as secondary products of cyanobacteria metabolism. They have a stable structure, and can bioaccumulate in living organisms. Humans and livestock who drink fresh water containing MC-LR can be poisoned. However, few studies have reported the effects of MC-LR exposure on livestock or human reproduction. In this study, we used porcine oocytes as a model to explore the effects of MC-LR on oocyte maturation, and studied the impact of vitamin C (VC) administration on MC-LR-induced meiosis defects. Exposure to MC-LR significantly restricted cumulus cell expansion and decreased first polar body extrusion. Further studies showed that MC-LR exposure led to meiosis arrest by disturbing cytoskeleton dynamics with MC-LR exposed oocytes displaying aberrant spindle organization, low levels of acetylate α-tubulin, and disturbed actin polymerization. Additionally, MC-LR exposure impaired cytoplasmic maturation by inducing mitochondria dysfunction. Moreover, MC-LR also produced abnormal epigenetic modifications, and induced high levels of oxidative stress, caused DNA damage and early apoptosis. The administration of VC provided partial protection from all of the defects observed in oocytes exposed to MC-LR. These results demonstrate that MC-LR has a toxic effect on oocyte meiosis through mitochondrial dysfunction-induced ROS, DNA damage and early apoptosis. Supplementation of VC is able to protect against MC-LR-induced oocyte damage and represents a potential therapeutic strategy to improve the quality of MC-LR-exposed oocytes.

## Introduction

The incidence of cyanobacteria blooms is increasing globally due to water eutrophication issues and global warming, and such blooms are now recognized as an emerging environmental threat (Huisman et al., 2018). Cyanobacterial blooms generate a secondary metabolite that can be highly toxic. Microcystins (MCs) are the most abundant and common cyanotoxins produced by toxic cyanobacteria and there has been a marked increase in the reports of human and livestock poisonings from the consumption of fresh water containing MCs (Roegner et al., 2014; Xue et al., 2016; Svircev et al., 2019). These cyanotoxins are characterized by their highly stable structure. They can bioaccumulate in aquatic animals owing to their ability to resist degradation under conditions found in most natural water ways (near-neutral pH) and are also resistant to high temperatures (Ait Abderrahim et al., 2019). Microcystin-leucine-arginine (MC-LR) is one of the most abundant and harmful microcystins (Machado et al., 2017; Wang et al., 2019). It is a potent hepatotoxin that has been linked to the development of primary liver cancer and is classified as a potential human carcinogen (Group 2B) by the International Agency for Research on Cancer (IARC) (Ueno et al., 1996; Zhao et al., 2016; Jia et al., 2019). A variety of toxicity tests have shown that MC-LR causes oxidative stress, and induces apoptosis and DNA damage, in mouse and human hepatocytes both *in vitro* and *in vivo* (Zhang et al., 2013; Ma et al., 2018; Chen et al., 2019; Wu et al., 2019). Accumulation of MC-LR has also been reported in reproductive organs, including the connective tissue of the ovary and the testis, as well as in oocytes (Qiao et al., 2019), and can severely impair the function of the reproductive system. In the zebrafish, MC-LR affected the endocrine system and oogenesis, and disrupted the meiotic maturation of oocytes *in vitro* (Trinchet et al., 2011; Zhao et al., 2015; Liu et al., 2016, 2019). MC-LR has also been reported to decrease sperm motility in male rats and induce toxic effects on Sertoli cells of the rat testis (Li et al., 2008; Chen et al., 2016). However, the effects of MC-LR on mammalian oocyte maturation and its possible mechanism have not yet been studied.

The maturity and quality of oocytes are important for fertilization and reproduction of mammals. During meiosis, precise regulation of spindle assembly and chromosomal organization are required to ensure the high developmental potential of oocytes. Spindle disorganization and chromosome misalignment will cause aneuploidy and cell cycle arrest (Xu et al., 2019), The proper spatial dynamics and normal functions of organelles in oocytes are also essential to ensure quality of oocytes. Particularly, when mitochondrial dysfunction occurs, the quality of oocytes is usually substandard (Babayev and Seli, 2015). Meanwhile, the level of ROS is also critical to oocyte maturation. Physiological level of ROS modulates oocyte functions, while its accumulation leads to oxidative stress and triggers apoptosis in oocytes (Tiwari et al., 2015). However, the environmental pollutants, including chemical, physical, and microbial pollutants, not only destroy the cytoskeleton and disturb the function of organelles of oocytes, but also cause epigenetic changes, which can lead to fetal developmental disorders and childhood diseases (Sun et al., 2020).

Vitamin C (L-ascorbic acid; VC) is a well-known antioxidant. It can donate electrons to reduce reactive oxygen species (ROS) and prevent damage to lipids, proteins and DNA during cell metabolism or from exposure to toxins and pollutants (Lykkesfeldt et al., 2014; Carr and Maggini, 2017; Cimmino et al., 2018). Studies have proved that VC is beneficial for mammalian reproduction. It can improve the development of preantral follicles during *in vitro* culture (Rossetto et al., 2009; Gomes et al., 2015), promote the meiotic maturation of pig oocytes (Tao et al., 2010; Yu et al., 2018), and improve the developmental competence of embryos after parthenogenetic activation and somatic cell nuclear transplantation (Kere et al., 2013). Furthermore, VC can ameliorate defects caused by environmental pollutants such as gamma-irradiation and heavy metal pollution in oocytes and embryos (Mozdarani and Nazari, 2009; Zhou et al., 2019b). Therefore, we hypothesized that VC could protect oocytes from defects induced by MC-LR during maturation.

Pigs share many physiological similarities with humans, and porcine cells are easier to obtain than human cells (Han et al., 2016), making porcine oocyte an ideal model to investigate human reproduction processes. Therefore, to investigate the toxic effects of MC-LR on mammalian oocyte and its possible mechanism, we used the porcine oocyte as a model. And we also analyzed the effects of VC on MC-LR exposure oocyte maturation, aimed to provide a potential therapeutic strategy to improve the quality of MC-LR-exposed oocytes.

## Materials and Methods

### Antibodies and Chemicals

Antibodies were as follows: Mouse monoclonal anti-α-tubulin antibody (1:200, Sigma, St. Louis, MO, United States, #F2168); anti-acetyl-α-tubulin (Lys-40) antibody (1:100, Sigma #T7451); phalloidin-TRITC (1:200, Sigma, #P1951); rabbit monoclonal to gamma H2A.X (gH2A.X) (1:200, Abcam, Cambridge, United Kingdom, #ab81299); rabbit polyclonal anti-di-methyl-histone H3 (Lys4) (H3K4me2) antibody (1:200, Cell Signaling Technology, Danvers, MA, United States); rabbit polyclonal anti-tri-methyl-histone H3 (Lys4) (H3K4me3) antibody (1:200, Cell Signaling Technology, United States, #C42D8); rabbit polyclonal anti-trimethyl-histone H3 (Lys36) (H3K36me3) antibody (1:200, ABclonal, United States, #A2366). Alexa Fluor 488 goat anti-mouse antibody (1:200, Invitrogen #A11126, Carlsbad, CA, United States); MitoTracker Red CMXRos (Thermo Fisher Scientific, #7512, Waltham, MA, United States); Alexa Fluor 594 goat anti-rabbit antibody (Invitrogen, Carlsbad, CA, United States). Microcystin-LR was purchased from APExBIO Technology (Houston, TX, United States, #B3698); VC was purchased from Sigma (Shanghai, China, #A7506). The basic maturation culture medium used was tissue culture medium (TCM-199; Sigma). Phosphate-buffered saline (PBS) was purchased from Life Technologies (Invitrogen).

### Oocyte Collection and *in vitro* Maturation (IVM)

Ovaries were obtained from a local slaughterhouse and transported to the laboratory in 0.9% NaCl containing 800 IU/ml of gentamicin at 37°C. *In vitro* oocyte maturation was performed as previously described (Li et al., 2009). Follicular fluid was collected from 3 to 8 mm follicles using an 18-gauge needle attached to a 10-ml disposable syringe. Cumulus–oocyte complexes (COCs) were then aspirated by vacuum suction from follicular fluid. After washing, the COCs with compact cumulus cells and a uniform ooplasm were selected to culture in 4-well dishes with *in vitro* maturation medium (IVM) [TCM-199 supplemented with 10% cattle serum (CS; Gibco), 10% (v/v) porcine follicular fluid, 0.8 mM L-glutamine, 75 mg/mL penicillin, 50 mg/mL streptomycin, 15 IU/mL pregnant mare serum gonadotropin (PMSG), and 15 IU/mL human chorionic gonadotropin (hCG)] at 38.5°C in an atmosphere of 5% CO_2_ with saturated humidity. After further culture for 26 h, COCs at MI stage were treated with hyaluronidase (1 mg/ml in TCM-199 culture medium) for approximately 1 min to obtain denuded oocytes (DOs). For oocytes at MII stage, they were collected after 44 h of culture and treated with hyaluronidase.

### MC-LR and VC Treatment

Microcystin-leucine-arginine was dissolved in TCM-199 culture medium and then in maturation medium to final concentrations of 20, 40, 80, and 120 μM. VC was dissolved in PBS and diluted with maturation medium to final concentrations of 50, 100, 200, and 500 μM. The VC was added to maturation medium immediately before use.

### Immunofluorescence Staining and Quantification

Denuded oocytes were collected and fixed in 4% paraformaldehyde (PFA) in PBS for 30 min at room temperature. After being washed for 15 min in wash buffer [Ca^2+^ and Mg^2+^-free PBS with 1% BSA (PB1)], oocytes were permeabilized in 1% Triton X-100 (in PBS) for 1 h at room temperature. Subsequently, they were blocked with PB1 for 1 h at room temperature to suppress the non-specific binding of IgG. DOs were then incubated with primary antibodies overnight at 4°C, washed three more times, and incubated with secondary antibody for 1 h at room temperature. Finally, Hoechst 33342 (10 μg/mL) was used to stain nuclei for 10 min at room temperature. Samples were mounted on glass slides and examined with a confocal laser-scanning microscope (LSM 700 META; ZEISS, Oberkochen, Germany). Image J software (version 1.46r, United States) was used for quantitative analysis. Negative controls were set using oocytes without the primary or secondary antibodies. When quantification of signal located in nuclear area, the average pixel from three different cytoplasm areas were used as background for normalization. For quantification of signal located in whole oocyte, the average pixel from five negative control oocytes was set as background for normalization. Finally, the net signal intensity of samples was performed using the average pixel intensity to subtract the background. At least 20 oocytes were analyzed in each group, and at least three replicates were performed for each experiment.

### Detection of Mitochondria and Reactive Oxygen Species (ROS)

A DCFH diacetate (DCFHDA) kit (Beyotime, China) was used to examine the level of intracellular ROS generated during oocyte maturation. Mito-Tracker Red CMXRos (Invitrogen, Eugene, OR, United States, #M7512) was used for mitochondria detection. After DOs were obtained, they were incubated in TCM-199 culture medium containing DCFHDA (1:800) or Mito-Tracker Red CMXRos (1:200) for 30 min at 38.5°C in a 5% CO_2_ incubator. After three washes in TCM-199, oocytes were placed on a glass slide and observed under the confocal laser-scanning microscope as soon as possible.

As for mitochondrial membrane potential (ΔΨm) detection, MitoProbe JC-1 Assay kits (M34152, Thermo Fisher Scientific, Waltham, MA, United States) was used. The steps of JC-1 staining are the same as above and 2 μM JC-1 was added in TCM-199 culture medium. The change in JC-1 from red (∼590 nm) to green (∼529 nm) fluorescence was used to detect a decrease in mitochondrial membrane potential. The ratio of red to green fluorescence intensity was analyzed using ImageJ software.

### Annexin-V Staining

Annexin-V staining kit (Vazyme, Nanjing, China) was used to detect early apoptosis level in oocytes. According to the manufacturer’s instruction, a total of 20–30 DOs at MI stage from each group were incubated in 100 μl binding buffer containing 10 μl of Annexin V-FITC for 10 min in the dark. Then oocytes were washed three times in D-PBS containing 0.1% BSA, placed on glass slides and observed under the confocal microscope immediately (ZEISS LSM 700 META, Germany).

### RNA Isolation and Real-Time Quantitative PCR

Porcine COCs maturated *in vitro* for 26 h, and the DOs in control group, MC-LR group, and VC-rescued group were then collected, respectively. Total RNA were extracted from 30 oocytes with a Dynabead mRNA DIRECT kit (Invitrogen Dynal, Oslo, Norway) according to the manufacturer’s instructions. First cDNA strand was synthesized using PrimeScript^TM^ RT Master Mix (Takara, Japan). Real-time Quantitative polymerase chain reaction (RT-qPCR) was performed using a fast real-time PCR system (ABI Step One Plus). Primer sequences are listed in Table 1. Gene expression levels were analyzed using the 2^–ΔΔ*Ct*^ method after the melting-curve analysis was completed and *GAPDH* was used as a control gene.

**TABLE 1:** Primer sequences for RT-qPCR.

**Gene**	**Forward primer**	**Reverse primer**
*ASH1L*	GCCTCTGACACGGACCC	TCCCGGCTACCAACAAGAGT
*NSD1*	GCTAGCTGCTTTCTACCCTGA	GCCAGCATCAACCGTGC
*MLL2*	TGGAAGTGCAAGTGGTGTGT	GTACGGGGCGTGACAGATAG
*SETD2*	CAGTCCGTCAGTGTACAGCA	GTCACAACCATTTCAGGTGGC
*KDM5B*	GACGTGTGCCAGTTTTGGAC	TCGAGGACACAGCACCTCTA
*KDM4A*	TGGAAAAGCAGTGGGATCGG	CTTTGGAGGAACAACCTTGGC
*GAPDH*	GATGGTGAAGGTCGGAGTG	CGAAGTTGTCATGGATGACC

### Statistical Analysis

At least three replicates were performed for each experiment. All analyses were performed using Graph Pad Prism 5 (Graph Pad Software Inc., San Diego, CA, United States) and were presented as mean percentages ± standard error of the mean (mean ± SEM). Inter-group differences were compared using *t*-tests. *p* values < 0.05 indicated statistical significance.

## Results

### VC Reduces Meiotic Defects in MC-LR-Exposed Oocytes

**FIGURE 1:**
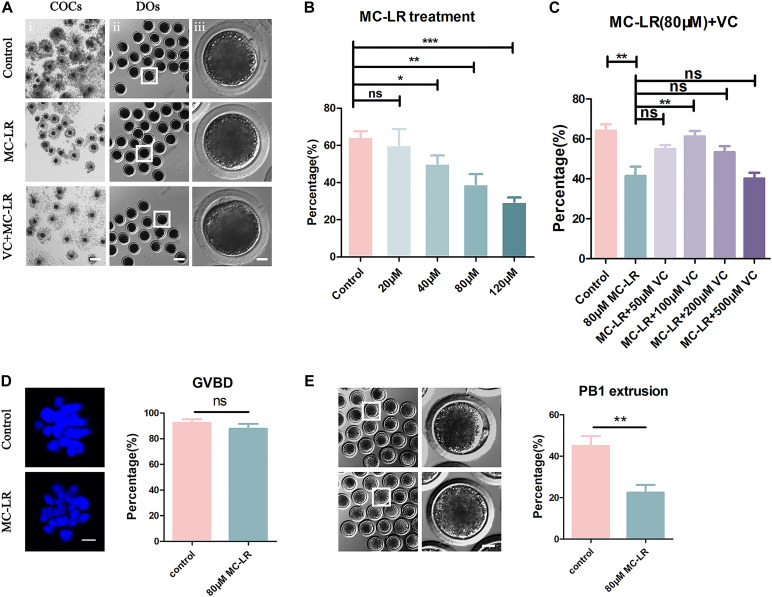
VC (100 μm) alleviates the meiotic defects in MC-LR-exposed oocytes. **(A)** Representative images of cumulus expansion and polar body extrusion (PBE) in the control, MC-LR-exposed, and VC-rescued groups (80 μM MC-LR and 100 μM VC were used). Bar = 150 μm **(i)**; 100 μm **(ii)**; 20 μm **(iii)**. **(B)** The rate of PBE was compared in control and different concentrations of MC-LR-exposed groups (20 μM, 40 μM, 80 μM, and 120 μM) after being cultured for 44 h *in vitro*. **(C)** The rate of PBE was recorded in control and different concentrations of VC-supplemented groups (50 μM, 100 μM, 200 μM, and 500 μM) after culture for 44 h with 80 μM MC-LR *in vitro*. **(D)** Representative images of chromosomes in porcine oocytes that underwent GVBD. And the rate of GVBD was recorded in control and MC-LR-exposed (80 μM) oocytes. Bar = 2.5 μm. **(E)** Representative images of PBE in the control and MC-LR-exposed (80 μM) DOs. Bar = 20 μm. * indicates significant difference at *p* < 0.05 level, ** indicates *p* < 0.01 level, *** indicates *p* < 0.001 level and ns indicates: no significant difference.

To explore the toxic effects of MC-LR exposure, oocytes were cultured with varying concentrations of MC-LR (0 μM, 20 μM, 40 μM, 80 μM, and 120 μM) for 44 h *in vitro*. The proportion of polar body extrusion (PBE) and COC viability in the control and MC-LR-exposed oocytes is shown in Figure 1. Almost all of the cumulus cells surrounding oocytes in the control group were fully expanded, while those in the MC-LR-exposed group exhibited poor expansion of COCs (Figure 1A). Moreover, the majority of oocytes in the control group reached the meiosis II (MII) stage after 44 h of culture, and demonstrated extrusion of the polar body, but exposure to MC-LR significantly reduced the PBE rate (control: 64.52 ± 3.11%, *n* = 131; 20 μM: 60.10 ± 8.71%, *n* = 108, *p* > 0.05; 40 μM: 50.18 ± 4.36%, *n* = 153, *p* < 0.05; 80 μM: 39.15 ± 5.39%, *n* = 152, *p* < 0.01; 120 μM: 29.63 ± 2.32%, *n* = 148, *p* < 0.001; Figure 1B). The concentration of 80 μM MC-LR was chosen for further studies because this not only caused obvious meiotic defects, but also allowed a proportion of oocytes to develop to the MII stage for further investigation.

Besides, we quantified the rate of the oocytes that underwent germinal vesicle breakdown (GVBD) following 80 μM MC-LR exposure and found that it was comparable with the controls (control: 92.2 ± 2.96%, *n* = 60, VS MC-LR: 86.59 ± 3.78%, *n* = 60, *p* > 0.05) (Figure 1D). This suggested that the meiotic arrest occurred after MI stage. Meanwhile, we checked the MC-LR effect on DOs (Figure 1E). 80 μM MC-LR significantly reduced the PBE rate in DOs (control: 44.94 ± 4.81%, *n* = 60, VS MC-LR: 22.49 ± 3.67%, *n* = 60, *p* < 0.01), suggesting that MC-LR worked directly on oocytes. However, considering the very low maturity rate of DOs, we used COCs for further investigation.

To investigate whether VC can alleviate meiotic arrest caused by MC-LR, VC was supplemented to the IVM culture medium containing 80 μM MC-LR. We found that 100 μM VC significantly increased the rate of PBE in MC-LR exposure oocytes compared with MC-LR alone (VC: 61.21 ± 2.17%, *n* = 150, VS MC-LR: 41.42 ± 4.7%, *n* = 145, *p* < 0.01) but the lower concentration of 50 μM or higher of 200 μM and 500 μM VC did not show the same effect (50 μM: 54.93 ± 2.03%, *n* = 109, *p* > 0.05; 200 μM: 53.35 ± 3.09%, *n* = 114, *p* > 0.05; 500 μM: 39.93 ± 2.68%, *n* = 115, *p* > 0.05) (Figures 1A,C). Thus, the concentration of 100 μM VC was chosen for further study. These results suggested that MC-LR exposure inhibited porcine oocyte maturation in a dose-dependent manner, but VC can protect oocytes against meiotic defects caused by MC-LR exposure.

### VC Alleviates Spindle Defects in MC-LR-Exposed Oocytes

**FIGURE 2:**
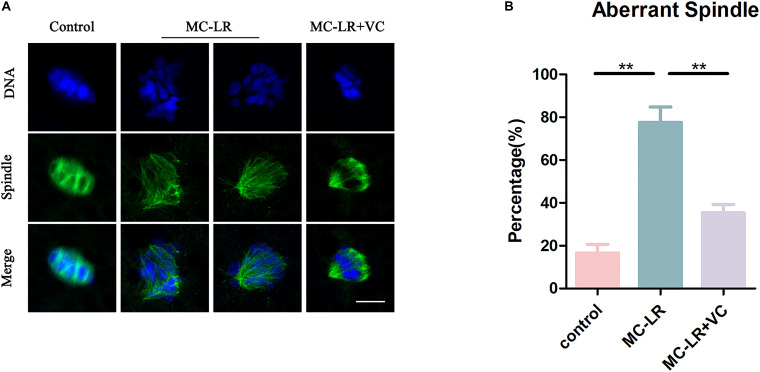
VC protects MC-LR-exposed oocytes from spindle defects. **(A)** Representative images of spindle morphology and chromosome alignment in the control, MC-LR-exposed, and VC-rescued groups. Bar = 5 μm. **(B)** The rate of aberrant spindles in the control, MC-LR-exposed, and VC-rescued groups. **p < 0.01.

Given that spindle formation is critical for PBE, we next examined spindle structures after MC-LR exposure. The results of immunofluorescence are shown in Figure 2. Most oocytes in the control group exhibited regular spindle morphology and good chromosome alignment on the equatorial plate. In contrast, spindle formation was severely disrupted, and the chromosomes were disorganized, in the MC-LR-exposed group. Quantitative analysis showed that MC-LR-exposed oocytes exhibited a significantly higher proportion of aberrant spindles than control oocytes (MC-LR: 77.58 ± 7.23%, *n* = 116, VS control: 16.57 ± 4.05%, *n* = 106, *p* < 0.01) (Figure 2B). However, supplementation of VC decreased the proportion of abnormal spindles caused by MC-LR exposure (VC: 35.27 ± 4.05%, *n* = 104, VS MC-LR: 77.58 ± 7.23%, *n* = 116, *p* < 0.01), indicating that VC can restore spindle defects in MC-LR-exposed oocytes.

### VC Restores α-Tubulin Acetylation Level in MC-LR-Exposed Oocytes

**FIGURE 3:**
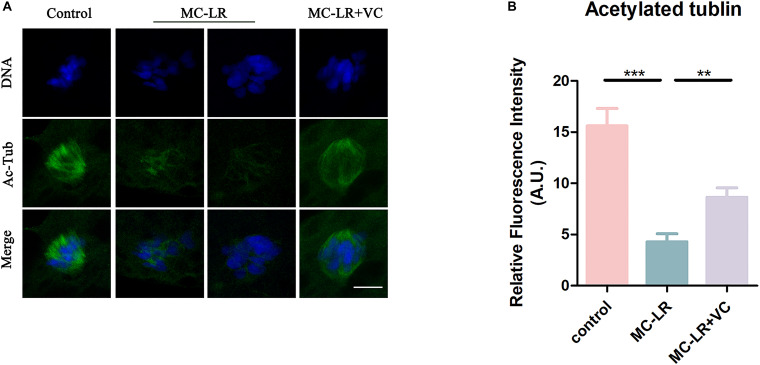
VC increases the level of acetylation of α-tubulin in MC-LR-exposed oocytes. **(A)** Representative images of α-tubulin in the control, MC-LR-exposed, and VC-rescued groups. Bar = 5 μm. **(B)** Quantitative analysis of the fluorescence intensity of acetylated α-tubulin in the control, MC-LR-exposed, and VC-rescued groups. **p < 0.01, ***p < 0.001.

We next examined the level of acetylated α-tubulin, through immunofluorescence, because this post-translational modification is critical for the maintenance of stable microtubules in both mitotic and meiotic cells. As shown in Figure 3, acetylated tubulin levels were significantly lower in MC-LR-exposed oocytes compared with control oocytes. Furthermore, VC supplementation significantly increased the level of acetylated α-tubulin in MC-LR-exposed oocytes. Quantitative analysis of the fluorescence intensity of acetylated α-tubulin validated these qualitative findings (MC-LR-exposed: 4.29 ± 0.79, *n* = 60, VS Control: 15.60 ± 1.71, *n* = 60, *p* < 0.001; VC supplementation: 8.64 ± 0.92, *n* = 60, VS MC-LR-exposed: 4.29 ± 0.79, *n* = 60, *p* < 0.01) (Figure 3B). These results suggested that MC-LR disordered spindle assembly by downregulating tubulin acetylation and that the presence of VC partly prevented these aberrations in oocyte development.

### VC Improves Actin Assembly of MC-LR-Exposed Oocytes

**FIGURE 4:**
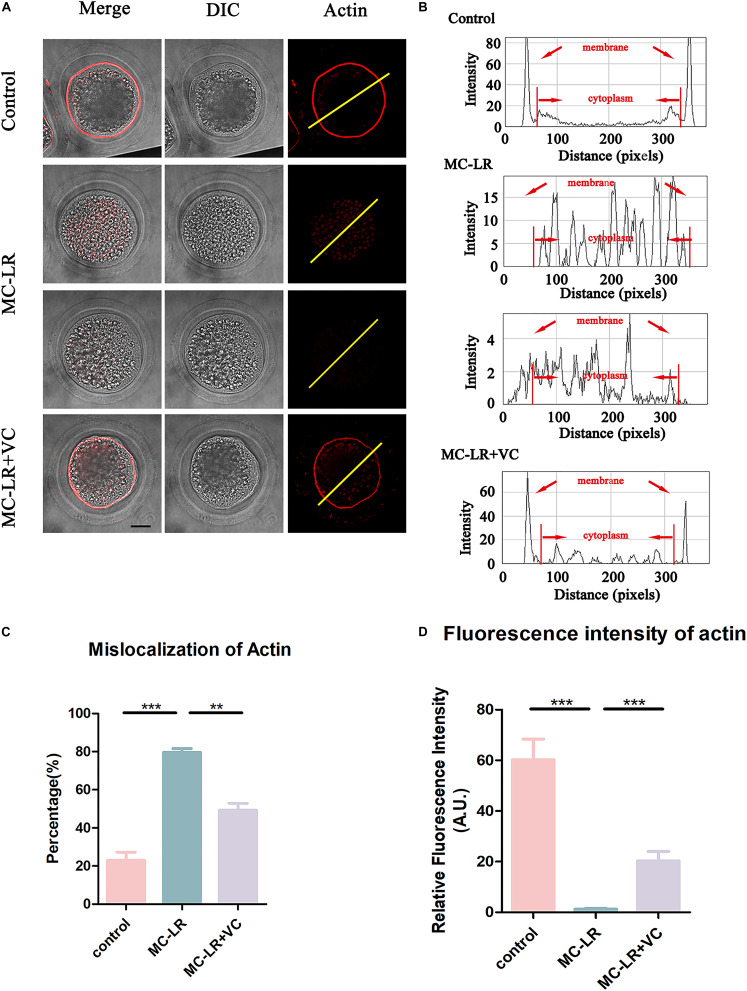
VC protects porcine oocytes from abnormal actin assembly caused by MC-LR exposure. **(A)** Representative images of F-actin distribution in the control, MC-LR-exposed, and VC-rescued groups. Bar = 20 μm. **(B)** Right graphs show fluorescence intensity profiling of phalloidin in oocytes. Lines were drawn through the oocytes, and pixel intensities were quantified along the lines. **(C)** The rate of mislocalization of actin. **(D)** Quantitative analysis of the fluorescence intensity of F-actin in the control, MC-LR-exposed, and VC-rescued groups. **p < 0.01, ***p < 0.001.

Because actin filaments are the main driving force for asymmetric division in mammalian oocytes, we next examined the actin assembly in both control oocytes and MC-LR-exposed oocytes. Phalloidin was used to label F-actin, and the results were shown in Figure 4. In the control group, the actin filaments in most oocytes were evenly accumulated at the cortical region and showed a strong immunofluorescent signal. However, in most MC-LR-exposed oocytes, the accumulation of F-actin at the cortical region significantly decreased (Figure 4A). The F-actin intensity from the lineation confirmed this (Figure 4B). Meanwhile, the proportion of mislocalized actin was significantly increased in MC-LR-exposed oocytes (MC-LR-exposed: 79.58 ± 2.00%, *n* = 96, VS Control: 22.93 ± 4.34%, *n* = 100, *p* < 0.001) and co-supplementation with VC supplementation significantly reduced actin abnormalities caused by MC-LR exposure (VC supplement: 49.27 ± 3.59%, *n* = 110, VS MC-LR-exposed: 79.58 ± 2.00%, *n* = 96, *p* < 0.01) (Figure 4C). Moreover, quantitative analysis of the F-actin fluorescence intensity (Figure 4D) also showed a significant decrease in MC-LR-exposed oocytes, compared with control group (MC-LR: 1.26 ± 0.34, *n* = 60, VS control: 60.24 ± 8.20, *n* = 60, *p* < 0.001). However, this was partially ameliorated by the co-supplementation of VC (VC: 20.30 ± 3.74, *n* = 60 VS MC-LR: 1.26 ± 0.34, *n* = 60, *p* < 0.001). These results showed that VC was able to partially protect porcine oocytes from abnormal actin assembly caused by MC-LR exposure.

### VC Reduces Mitochondrial Abnormalities in MC-LR-Exposed Oocytes

**FIGURE 5:**
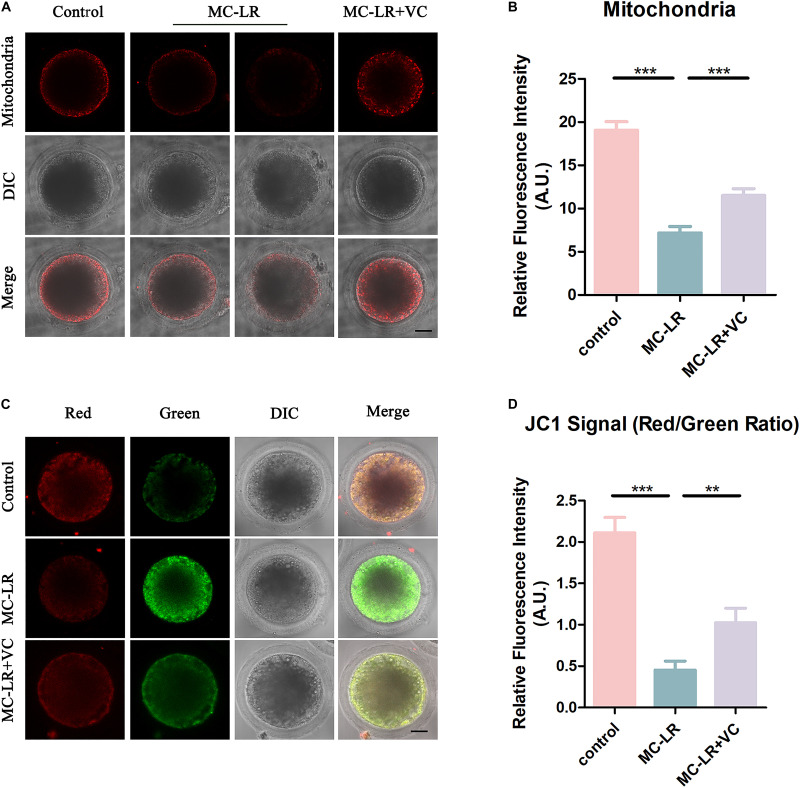
VC protects MC-LR-exposed oocytes from mitochondrial damage. **(A)** Representative images of mitochondria distribution in the control, MC-LR-exposed, and VC-rescued groups. Bar = 20 μm. **(B)** Quantitative analysis of the fluorescence intensity of mitochondria. **(C)** Mitochondrial membrane potential (ΔΨm) was detected by JC-1 in control, MC-LR-exposed, and VC-rescued groups (Red, high ΔΨm; Green, low ΔΨm). Bar = 20 μm. **(D)** The ratio of red and green fluorescence intensity was recorded in control, MC-LR-exposed, and VC-rescued groups. **p < 0.01, ***p < 0.001.

The mitochondrion is essential for oocyte cytoplasmic maturation, and is a primary organelle that supplies the majority of the cellular ATP for oocyte maturation (Perez et al., 2000; Zhou et al., 2019a). To determine whether MC-LR exposure caused abnormal mitochondria, we used MitoTracker Red CMXRos to label mitochondria, and the results are shown in Figure 5. In control oocytes, the mitochondria signals were mainly seen in the subcortical regions around lipid droplets. The exposure of oocytes to MC-LR resulted in an abnormal pattern of mitochondrial distribution (Figure 5A). Quantitative fluorescence intensity analysis showed that the mitochondrial signals were reduced in MC-LR-exposed oocytes compared with the control group (MC-LR: 7.18 ± 0.74, *n* = 60, VS control: 19.06 ± 0.98, *n* = 60, *p* < 0.001) (Figure 5B). Supplementation of VC to MC-LR-exposed oocytes caused the distribution of mitochondria in these samples to appear similar to the control group and increased the fluorescent intensity of the mitochondrial signal compared with MC-LR-exposed oocytes which did not receive VC supplementation (VC: 11.50 ± 0.80, *n* = 60, VS MC-LR: 7.18 ± 0.74, *n* = 60, *p* < 0.001) (Figure 5).

To further study the effect of MC-LR on mitochondrial function, we also assessed the mitochondrial membrane potential (ΔΨm) by JC-1 staining (Figures 5C,D). Mitochondria with high membrane potential showed a red fluorescence while those with low membrane potential showed a green fluorescence. Quantitative analysis revealed that the ratio of red to green fluorescence was significantly higher in the control group than in MC-LR-exposed oocytes (control: 2.11 ± 0.19, *n* = 30, VS MC-LR: 0.45 ± 0.10, *n* = 30, *p* < 0.001). But rescued in VC-rescued groups (MC-LR: 0.45 ± 0.10, *n* = 30, VS VC: 1.03 ± 0.18, *n* = 30, *p* < 0.01). Taken together, these results suggested that VC prevented mitochondrial dysfunction induced by MC-LR in oocytes.

### VC Restores Abnormal Epigenetic Alterations in MC-LR-Exposed Oocytes

**FIGURE 6:**
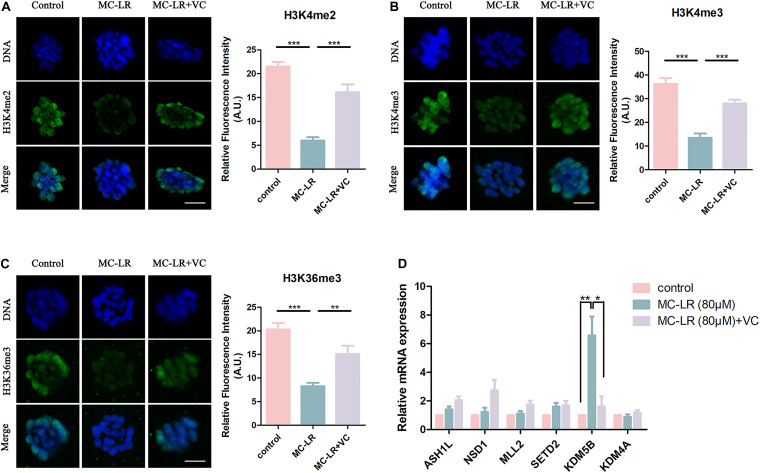
VC can protect oocytes from abnormal epigenetic alterations caused by MC-LR exposure. **(A–C)** Representative images of H3K4me2, H3K4me3, and H3K36me3 in the control, MC-LR-exposed, and VC-rescued groups. Green, H3K4me2, H3K4me3, or H3K36me3; Blue, chromosomes. Bar = 5 μm. Quantitative analysis of the fluorescence intensity of H3K4me2, H3K4me3, and H3K36me3 were recorded in graphs. **(D)** Relative mRNA levels of methyltransferases and demethylases related to histone methylation markers (*ASH1L*, *NSD1*, *MLL2*, *SETD2*, *KDM5B*, and *KDM4A*). *Indicates significant difference at *p* < 0.05 level, **p < 0.01 level, and ***p < 0.001 level.

Histone methylation modification is a pivotal epigenetic modification that is critical for the regulation of gene expression and gene silencing. Disruption of histone modifications in the oocyte causes defective chromosome condensation and segregation, delayed maturation progression, and even oocyte aging (Gu et al., 2010; Qian et al., 2015). The level of histone H3 lysine 4 di-methylation (H3K4me2), histone H3 lysine 4 tri-methylation (H3K4me3), and histone H3 lysine 36 tri-methylation (H3K36me3) were studied to assess potential epigenetic modifications in MC-LR-exposed oocytes. As shown in Figure 6A, the fluorescence intensities of H3K4me2, H3K4me3, and H3K36me3 were significantly decreased in MC-LR-exposed oocytes compared with the control group. However, VC supplementation alleviated these defects to some extent (Figures 6A–C). Quantitative analysis also confirmed this (H3K4me2: MC-LR-exposed: 6.012 ± 0.69, *n* = 60, VS Control: 21.53 ± 0.94, *n* = 60, *p* < 0.001; VC supplement: 16.14 ± 1.61, *n* = 60, VS MC-LR-exposed: 6.012 ± 0.69, *n* = 60, *p* < 0.001. H3K4me3: MC-LR-exposed: 13.47 ± 1.89, *n* = 60, VS Control: 36.25 ± 2.52, *n* = 60, *p* < 0.001; VC supplement: 28.00 ± 1.62, *n* = 60, VS MC-LR-exposed: 13.47 ± 1.89, *n* = 60, *p* < 0.001. H3K36me3: MC-LR-exposed: 8.25 ± 0.75, *n* = 60, VS Control: 20.31 ± 1.35, *n* = 60, *p* < 0.001; VC supplement: 15.09 ± 1.77, *n* = 60, VS MC-LR-exposed: 8.25 ± 0.75, *n* = 60, *p* < 0.01).

Then we examined the mRNA expression levels of H3K4me2, H3K4me3, and H3K36me3 methyltransferases (*ASH1L*, *MLL2*, *NSD1*, and *SETD2*) and demethylases (*KDM5B* and *KDM4A*) (Figure 6D). We found significant increase of *KDM5B* mRNA in MC-LR-exposed oocytes (MC-LR: 5.56 ± 1.08 VS control: 1, *p* < 0.01). But the mRNA expression levels of other five genes did not significantly differ from that of control group. However, VC supplementation significantly decreased the high mRNA expression level of *KDM5B* caused by MC-LR exposure (VC: 1.62 ± 0.70 VS control: MC-LR: 5.56 ± 1.08, *p* < 0.05). These results suggested that MC-LR led to abnormal histone methylations by affecting their corresponding demethylase *KDM5B*. But VC can protect oocytes from abnormal epigenetic alterations caused by MC-LR exposure.

### VC Decreases Oxidative Stress, DNA Damage and Apoptosis in MC-LR-Exposed Oocytes

**FIGURE 7:**
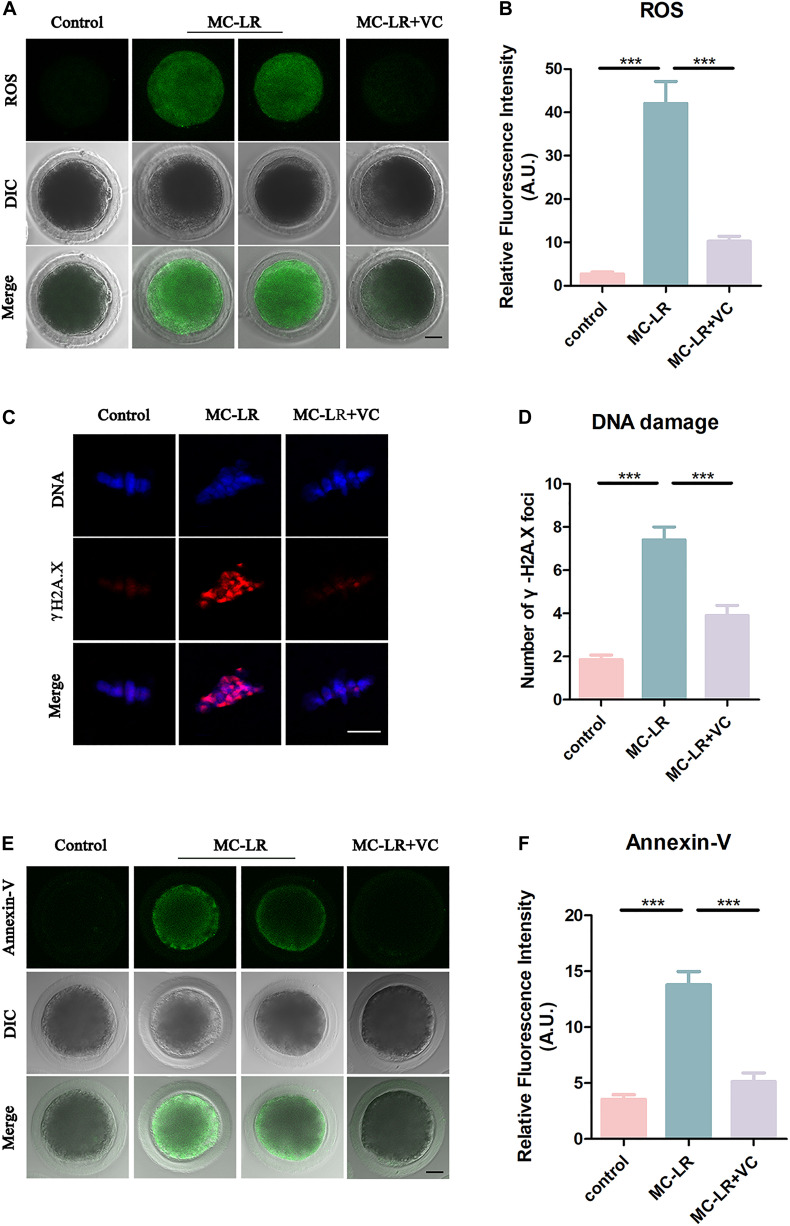
VC decreases oxidative stress, alleviates DNA damage, and rescues early apoptosis in MC-LR-exposed oocytes. **(A)** Representative images of ROS level in the control, MC-LR-exposed, and VC-rescued groups. Bar = 20 μm. **(B)** Quantitative analysis of the fluorescence intensity of ROS. **(C)** Immunofluorescent staining of γ-H2A.X showing DNA damage in control, MC-LR-exposed, and VC-rescued groups. Bar = 5 μm. **(D)** Quantification of the numbers of γ-H2A.X foci per oocyte in control, MC-LR-exposed, and VC-rescued groups was analyzed (total number of oocytes analyzed: *n* = 20 for control; *n* = 20 for MC-LR-exposed, *n* = 20 for VC-rescued). Data are expressed as mean ± SEM from three independent experiments. **(E)** Representative images of apoptotic oocytes in the control, MC-LR-exposed, and VC-rescued groups. Bar = 20 μm. **(F)** Quantitative analysis of the fluorescence intensity of Annexin-V. ***p < 0.001.

Microcystin-leucine-arginine induces cytotoxicity via oxidative stress in many kinds of cells, including tissues of the ovary (Li et al., 2008; Liu et al., 2018). To investigate whether MC-LR was inhibiting oocyte maturation via oxidative stress, we used DCFH staining to compare the ROS levels between control and MC-LR-exposed oocytes. MC-LR exposure resulted in increased ROS levels compared with oocytes in control group (MC-LR: 42.06 ± 5.09, *n* = 60, VS control: 2.73 ± 0.46, *n* = 60, *p* < 0.001; Figure 7A) and VC significantly reduced the excessive ROS present in MC-LR-exposed oocytes (VC: 10.28 ± 1.16, *n* = 60, VS MC-LR: 42.06 ± 5.09, *n* = 60, *p* < 0.001) (Figures 7A,B). These results suggested that VC decreased oxidative stress caused by MC-LR exposure during oocyte maturation.

Because oxidative stress can damage DNA, and MC-LR has been reported to inhibit DNA repair (Lankoff et al., 2006), we examined DNA damage by γ-H2A.X staining. Remarkably, we found that γ-H2A.X foci in MC-LR-exposed oocytes were significantly increased as compared to control oocytes (MC-LR: 7.4 ± 0.6, *n* = 20, VS Control: 1.85 ± 0.22, *n* = 20, *p* < 0.001) (Figures 7C,D), whereas VC supplement significantly reduced the γ-H2A.X foci (VC: 3.9 ± 0.47, *n* = 20, VS MC-LR: 7.40 ± 0.61, *n* = 20, *p* < 0.001). These results suggested that VC can protect oocytes from DNA damage caused by MC-LR exposure.

Since the excessive oxidative stress always induces apoptosis, we also assessed early apoptosis in oocytes by Annexin-V staining. The immunofluorescence results showed that the fluorescent signals of Annexin-V were rarely present in control oocytes, but significantly arose in MC-LR-exposed oocytes (MC-LR: 13.79 ± 1.19, *n* = 30, VS Control: 3.53 ± 0.42, *n* = 30, *p* < 0.001) (Figures 7E,F). However, early apoptotic signals were decreased in VC-rescued oocytes (VC: 5.13 ± 0.78, *n* = 30, VS MC-LR: 13.79 ± 1.19, *n* = 30, *p* < 0.001). Taken together, our results indicated that MC-LR induced early apoptosis in porcine oocytes while VC alleviated this defect.

## Discussion

The United States, China, Japan, and Europe have reported MC-LR contamination of freshwater resources and the frequency of these reports is increasing worldwide (Lee et al., 2017). The cyanotoxin can bioaccumulate in living organisms and its chemical structure is highly stable during cooking, and resistant to chemical breakdown (Ait Abderrahim et al., 2019). Humans and livestock living near freshwater bodies contaminated with cyanobacteria can be poisoned by drinking water containing MC-LR. However, the current studies on MC-LR reproductive toxicity are predominately focused on fish. Few studies have reported the effects on mammalian reproduction, including that of humans. To investigate the effects of MC-LR on mammalian reproductive function, we used porcine oocytes as a model because of their similar physiology to human oocytes. Our results showed that appropriate doses of VC were able to protect against abnormalities to cytoskeleton dynamics, mitochondrial distribution, epigenetic modification, oxidative stress and early apoptosis caused by MC-LR.

Given that cumulus cell expansion and the first PBE rate are two critical biological events occurring during meiotic progression (Zhang X. et al., 2019), we first examined these two important events and found poor expansion of COCs and a depressed PBE rate in MC-LR-exposed oocytes. This indicated that oocyte maturation was retarded by MC-LR exposure in a dose-dependent manner. The supplementation of VC with MC-LR partially protected oocytes from the negative effect of MC-LR on COC expansion and PBE during oocyte maturation. However, high concentration of VC actually caused a decrease in PBE rate. This may be because high level of VC acts as a pro-oxidant (El Banna et al., 2019) and will cause cytotoxicity to oocytes.

To further investigate how MC-LR exposure causes meiosis failure, we next examined cytoskeleton dynamic coordination. The results showed that spindle morphology and actin distribution in oocytes were severely disrupted by MC-LR. Moreover, the acetylation level of α-tubulin, which is a marker of spindle stability (Miao et al., 2018), was significantly reduced. These results are consistent with previous studies that MC-LR exposure induced microfilament and microtubule alterations, and caused the progressive disassembly of actin fibers in many kinds of cells (Ding et al., 2001; Beyer et al., 2012; Huang et al., 2015). Besides cytoskeleton dynamics, epigenetics modification is also a critical event for oocyte maturation (Gu et al., 2010). In this study, we examined histone methylation levels and found a significantly decrease in MC-LR-exposed oocytes. Meanwhile, the demethylase *KDM5B* mRNA level was significantly changed by MC-LR exposure, indicating that MC-LR meiotic maturation failure may result from an impairment of epigenetic modifications in porcine oocytes. These results consistent with the opinion which indicated that pollutants might cause cell dysfunctions via epigenetic modifications (Genchi et al., 2020; Zheng et al., 2020). However, VC restored these abnormal epigenetic alterations in MC-LR-exposed oocytes. This confirmed that VC can act as an epigenetic regulator to enhance cell functions (Zhang T. et al., 2019). Moreover, considering that changes in ATP levels correlate with the dysfunction of mitochondria present in MC-LR-exposed oocytes (Yu et al., 2010), we concluded that disturbed cytoskeleton, abnormal epigenetics, and dysfunctional mitochondria were the reasons for the meiosis failure in MC-LR-exposed oocytes.

We then tried to further explore the possible mechanism for the toxicity of MC-LR on oocytes. Studies have found that MC-LR caused cytotoxicity through mitochondrial signaling pathway. Mitochondrial dysfunction can induce an increase of ROS, whereas excessive ROS always caused DNA damage and early apoptosis in cells (Roth, 2018). Due to our results about the dysfunctional mitochondria caused by MC-LR exposure, we next examined oxidative stress in MC-LR-exposed oocytes. Similar with previous studies, high levels of ROS, DNA damage and apoptosis were found after MC-LR exposure. However, all these defects were rescued by VC supplementation. Given that increased levels of ROS have previously been associated with cytoskeletal disorganization, cell cycle arrest in human oocytes, and induced the change of epigenetic regulation (Prasad et al., 2016; Tiwari et al., 2017; Walters et al., 2018). We came to the conclusion that VC prevented mitochondrial dysfunction-induced oxidative stress and early apoptosis, which further affected epigenetic modifications and cytoskeleton dynamics in porcine oocytes, finally rescued meiosis defects caused by MC-LR exposure. However, because of the limited number of cells, researches on oocytes become difficult. Therefore, more studies still need to further explore the molecular mechanism of MC-LR toxicity and the protective role of VC on oocyte maturation.

## Conclusion

Taken together, our results indicate that the presence of MC-LR is deleterious to the maturation of porcine oocytes. The cyanotoxin generates mitochondrial dysfunction-induced ROS, causes DNA damage and induces early apoptosis, which further affects epigenetic modifications and cytoskeleton dynamics in porcine oocytes. Supplementation with VC reduces the severity of MC-LR-induced cell defects and provides a potential therapeutic strategy to improve the quality of MC-LR exposed oocytes.

## Data Availability Statement

The original contributions presented in the study are included in the article, further inquiries can be directed to the corresponding author.

## Ethics Statement

The animal study was reviewed and approved by the Care and Use of Laboratory Animals prepared by the Institutional Animal Care and Use Committee of Nanjing Agricultural University, China.

## Author Contributions

All authors were involved in designing and planning the experiments, preparing and reviewing the article. XZ, CZ, and WL performed the experiments. XZ and HL analyzed the data and wrote the article.

## Conflict of Interest

The authors declare that the research was conducted in the absence of any commercial or financial relationships that could be construed as a potential conflict of interest.
